# Production of Synthetic Lightweight Aggregates from Industrial Sludge

**DOI:** 10.3390/ma15124097

**Published:** 2022-06-09

**Authors:** How-Ji Chen, Pen-Chou Chen, Ching-Fang Peng, Chien-Wei Huang

**Affiliations:** Department of Civil Engineering, National Chung-Hsing University, No. 250, Kuo Kuang Road, Taichung 402, Taiwan; hojichen@nchu.edu.tw (H.-J.C.); s883309kimo@gmail.com (P.-C.C.); cute1309@gmail.com (C.-F.P.)

**Keywords:** lightweight aggregate, industrial sludge, water treatment sludge, waste recycling

## Abstract

Industrial sludge continues to increase in quantity with the development of industry. Therefore, how to effectively treat industrial sludge continues to be an environmental focus around the world. Due to the high calorie content of industrial sludge in Taiwan, most of the sludge is incinerated for simplicity and convenience. However, this incineration causes environmental pollution and cannot effectively reuse the industrial sludge. In this study, we investigated the feasibility of lightweight aggregates produced from water treatment sludge (WTS) mixed with industrial sludge. The industrial sludge was obtained from three industrial zones in Taiwan. The lightweight aggregate materials were prepared by mixing WTS with 7.5%, 15.0%, or 22.5% of industrial sludge as a secondary ingredient. The chemical composition analyses revealed that the ternary phase diagrams of the chemical components were within the range recommended by C.M. Moreover, Riley indicated that the ingredients could expand. The sintering experiments were conducted in two stages. Stage I served to determine the optimal sintering temperature, while Stage II produced lightweight aggregates at the optimal sintering temperatures after 5, 10, or 15 min of preheating. The results indicated that an increase in preheating time increased the particle density of the lightweight aggregates but decreased the water absorption ratio, because increasing preheating time causes more gases to escape, resulting in aggregates that could not produce sufficient gas to support expansion during the sintering stage. The sintering results of the lightweight aggregates showed that their particle density was between 0.5 and 1.4 g/cm^3^, which met the requirements of the specification. Their water absorption rate was below 21%, which was roughly in line with the recommendations of the specification. When the amount of industrial sludge added was less than 22.5%, the lightweight aggregate was sintered successfully, which is suitable for engineering applications. The industrial sludge experienced a loss of between 50% and 70% on ignition, resulting in pores and cracks that were observed on the surfaces of the lightweight aggregate. Based on the energy required to sinter lightweight aggregates, a greater loss on ignition indicates the conversion of more materials into heat energy. Therefore, the use of industrial sludge in lightweight aggregates not only provides ways to reuse industrial sewage waste but also reduces the consumption requirements for sintering lightweight aggregates, thereby achieving energy-saving and carbon-reduction goals.

## 1. Introduction

The application of lightweight aggregates has been around for almost a hundred years. In addition to being used as civil engineering materials such as structural aggregates [1,2], masonry aggregates, and non-structural aggregates [3], lightweight aggregates can also be used as horticultural and filter materials. Lightweight aggregates produced in the early days were mostly derived from natural materials. The production of artificial lightweight aggregates is shifting toward the reuse of resources. In addition to industrial byproducts (e.g., slag and fly ash), sewage and other types of waste sludge have also been used as production raw materials [4,5,6,7]. In our previous research results, reservoir sludge, paper sludge, and water treatment sludge (WTS) were successfully sintered to produce lightweight materials. The present study is a continuation of our prior research and further explores the feasibility of producing lightweight aggregates by using sludge from industrial zones in Taiwan [8,9,10].

Industrial wastewater sludge is waste produced by industrial wastewater treatment plants in industrial zones. Taiwan has 60 industrial zones and 39 wastewater treatment plants that treat wastewater discharge. The chemical composition of industrial sludge varies according to the properties of the wastewater, the method of wastewater treatment, and the operational practices implemented by a wastewater treatment plant. Because this type of sludge is classified as commercial waste, it cannot be discarded at will; therefore, the recycling of wastewater sludge from industrial zones is increasing as a trend. At present, industrial wastewater sludge in Taiwan is mostly reused for farmland composting and soil improvement. However, the complexity of industrial wastewater sources increases the complexity of the composition of industrial sludge. As such, the objective of the present study was to investigate the feasibility of sintered lightweight aggregates made with industrial wastewater sludge.

WTS is the byproduct of the solid–liquid separation process in water treatment plants. The chemical composition of WTS varies across Taiwan, depending on the quality of raw water and the types of coagulants that are used. The two types of coagulants that are currently used consist of either iron or aluminum salts, resulting in varying levels of aluminum and iron in the sludge. The other parts of the chemical composition in WTS do not present a significant difference since the water sources do not obviously vary in Taiwan. WTS is currently reused as a raw material for cement and red bricks, potting soil, and construction materials [11,12], and it can also be sintered to produce lightweight aggregates [13,14]. Huang and Wang collected sludge from 10 water treatment plants in Taiwan and successfully sintered the collected samples to product lightweight aggregates at a preheating temperature of 500 °C and various sintering temperatures [15].

The present study began with the analysis of the chemical composition of WTS and industrial sludge to determine the feasibility of sintered sludge forming lightweight aggregates. Next, based on the chemical composition levels that are recommended in the literature, the raw materials of lightweight aggregates were prepared by mixing varying proportions of WTS with industrial sludge. Thereafter, Stage I sintering experiments were performed, and the particle density and water absorption of the lightweight aggregates were analyzed to determine the optimal sintering temperature. Stage II sintering experiments were performed at the determined optimal sintering temperature. The feasibility of sintering industrial sludge into lightweight aggregates was determined on the basis of the particle density, water absorption, and loss on ignition.

## 2. Experimental Materials and Methods

WTS and industrial sludge were used as the primary and secondary raw materials, respectively; the raw materials in the present study were sourced from northern (denoted as A), southern (denoted as B), and central (denoted as C) industrial zones in Taiwan. An X-ray fluorescence (XRF) study was conducted to analyze the chemical compositions of the WTS and industrial sludge. The results indicated that the collected industrial sludge could not be expanded into lightweight material, and it could only be added as a secondary raw material in lightweight aggregate. On the basis of the chemical composition analysis, the industrial sludge was added at percentages of 7.5%, 15.0%, and 22.5% according to weight. Dried WTS and industrial sludge were pulverized by a crusher and then blended with water in a mixer until uniform to create 12 mm round granules, which were then placed in a drying oven and dried at 105 °C for 24 h.

**Figure 1:**
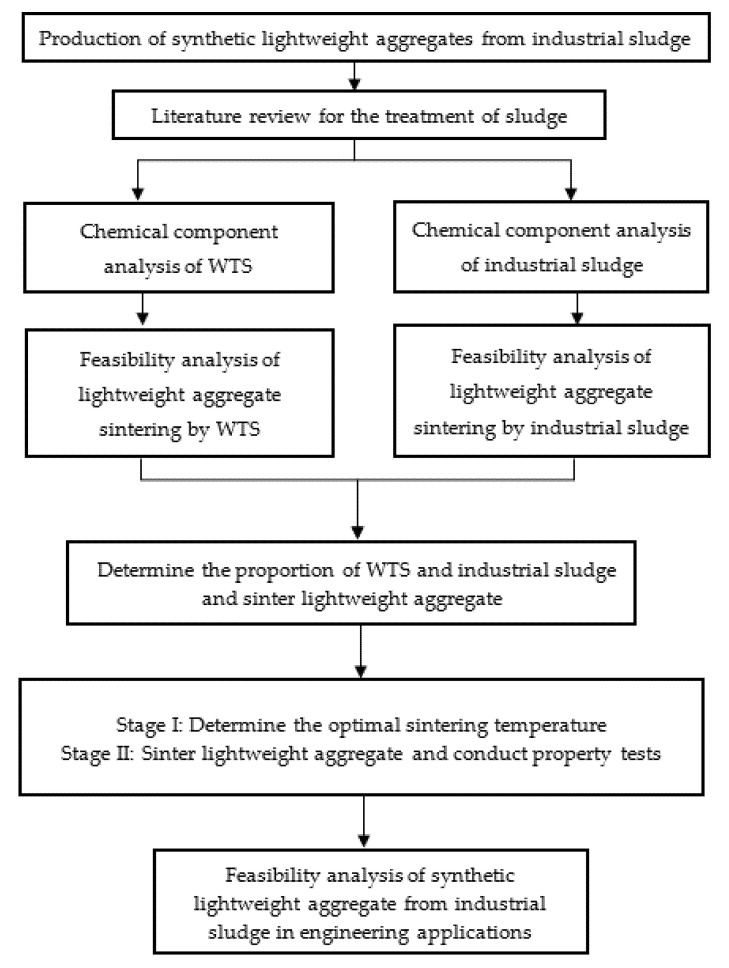
Experimental design and research flowchart.

In the present study, the dried aggregates were placed in an alumina saggar for sintering by a two-stage rapid heating process. Preheating and sintering were performed in two separate small and programmable high-temperature furnaces. Based on the results of previous studies [9,15], the Stage I sintering experiments were performed at a preheating temperature of 500 °C and sintering temperatures of between 1125 and 1200 °C for 12.5 min. The Stage II sintering experiments were performed at the same preheating temperature of 500 °C, and the sintering temperature was selected from the optimal sintering temperatures determined by the Stage I results for the same duration of 12.5 min. The sintering temperature of the second stage was determined according to the particle density of the sintered lightweight aggregate in the first stage being less than 1.0 g/cm^3^. The sintering conditions for both stages are presented in Table 1. Figure 1 presents the experimental design and research flowchart.

## 3. Results and Discussion

### 3.1. Composition Analysis of Waste Sludge

SiO_2_ in a sludge composition affects the glassy viscosity during the sintering of lightweight aggregates. A higher SiO_2_ content increases the glassy viscosity of the aggregates but reduces their strength. Al_2_O_3_ affects the sintering temperature as an increased content increases the sintering temperature. SiO_2_, Al_2_O_3_, and Fe_2_O_3_ are the main components of the glassy melt produced in the aggregates under high temperatures, and the contents of these three components must together comprise approximately 75% or more of the raw material by weight. When the contents of these three components are excessively low, the smoothness and strength of the sintered aggregates are affected. However, when the contents are excessively high, the melting point and viscosity of the sintered aggregates increase, and the expansion is poorer [16,17,18]. Table 2 lists the compositions of the sludge samples, obtained through an XRF analysis. Table 3 reveals that the total content of SiO_2_, Al_2_O_3_, and Fe_2_O_3_ was 84.77% in the WTS, suggesting that WTS can be sintered into lightweight aggregates with a smooth surface [13,14,15]. The total contents of these components (17.73%, 15.57%, and 11.59% in Samples A, B, and C, respectively) were considerably less than the recommended level of 75% for the industrial sludge samples; thus, the samples could not produce a glassy melt and be sintered into lightweight aggregates. Consequently, the industrial sludge was only considered as a secondary raw ingredient in the present study.

### 3.2. Evaluation of Waste Sludge as a Raw Material for Lightweight Aggregates

The chemical components Fe_2_O_3_, K_2_O, Na_2_O, CaO, MgO, and FeO are fluxing oxides that can reduce the temperature required to sinter lightweight aggregates. K_2_O and Na_2_O have the strongest fluxing ability, followed by CaO, MgO, and FeO. Fluxing agents increase the formation of the molten liquid phase and reduce the viscosity of materials at high temperatures. The contents of CaO, MgO, and FeO should not be excessively high; otherwise, the sintering temperature range is narrowed, reducing the expansion of the aggregates. Therefore, having set amounts of K_2_O and Na_2_O in the raw materials yields more favorable fluxing effects [18]. Table 4 indicates that the levels of fluxing agents were 15.32% in WTS, 12.37% in Sample A, 10.95% in Sample B, and 8.98% in Sample C. The industrial sludge samples A, B, and C had insufficient K_2_O and Na_2_O levels; thus, the industrial sludge in this study could not be independently used as raw materials for sintering lightweight aggregates.

The SiO_2_ and Al_2_O_3_ components in the raw materials must be melted under high temperatures, and the mass ratios of these components to the fluxing agents can be employed to estimate the softening temperature during the sintering of lightweight aggregates. When the chemical composition (SiO_2_ + Al_2_O_3_)/fluxing ratio is between 3.5 and 10, the raw materials are more likely to expand [18,19]. When the mass ratio is less than 3.5, the glassy viscosity of the raw materials becomes excessively low, such that expanding gases cannot be enveloped, resulting in aggregates exhibiting poorer foaming and expansion performance. When the mass ratio is greater than 10, the sintering temperature must be increased [20,21].

Based on the chemical composition analysis of each raw material, WTS was selected as the primary component of lightweight aggregates in the present study, and industrial sludge was added as a secondary component at 7.5%, 15.0%, and 22.5%. Table 5 presents the analysis results of the (SiO_2_ + Al_2_O_3_)/fluxing ratio for WTS and industrial sludge. The number in the sample name represents the percentage of industrial sludge. For example, A7.5 indicates Sample A with 7.5% industrial sludge and 92.5% WTS. The result demonstrates that except for A22.5, B22.5, and C22.5, which had mass ratios that were slightly lower than 3.5, the recorded mass ratios were between 3.5 and 10 and are therefore within the range recommended by the literature.

**Figure 2:**
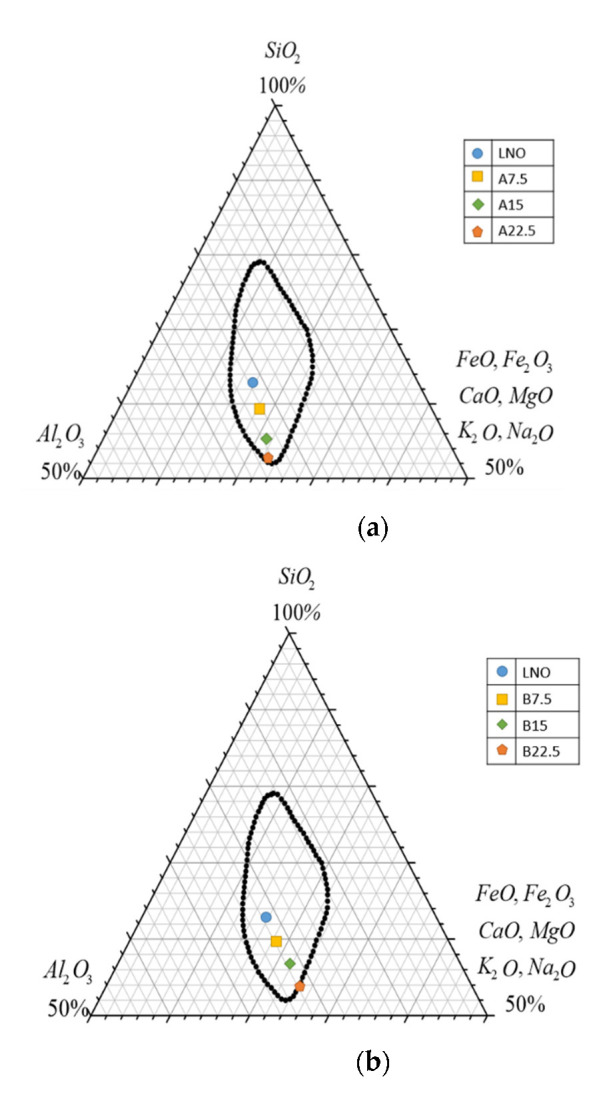

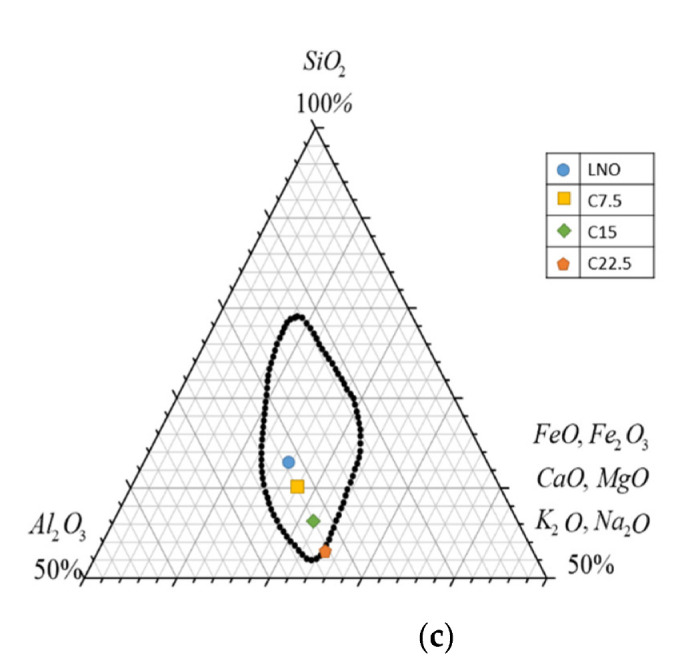
Ternary phase diagram of water treatment sludge mixed with (**a**) Industrial Sludge A, (**b**) Industrial Sludge B, and (**c**) Industrial Sludge C.

The ternary phase diagram developed by C.M. Riley is the most accepted theory for the sintering of lightweight aggregates. Figure 2 illustrates C.M. Riley’s ternary phase diagram based on the contents of SiO_2_, Al_2_O_3_, and the fluxing oxides; the circled section of the figure indicates the range that is conducive to the expansion of lightweight aggregates [16,22]. The figure demonstrates that for Samples A, B, and C, when the proportion of industrial sludge increases, the points in the figure begin to move gradually beyond the expansion range. When the proportion of industrial sludge is 22.5%, the point is on the boundary of the expansion range. Hence, according to C.M. Riley theory, every combination of WTS and industrial sludge considered in this study can be sintered into lightweight aggregates.

### 3.3. Results of Stage I Sintering of Industrial Sludge Lightweight Aggregates

**Figure 3:**
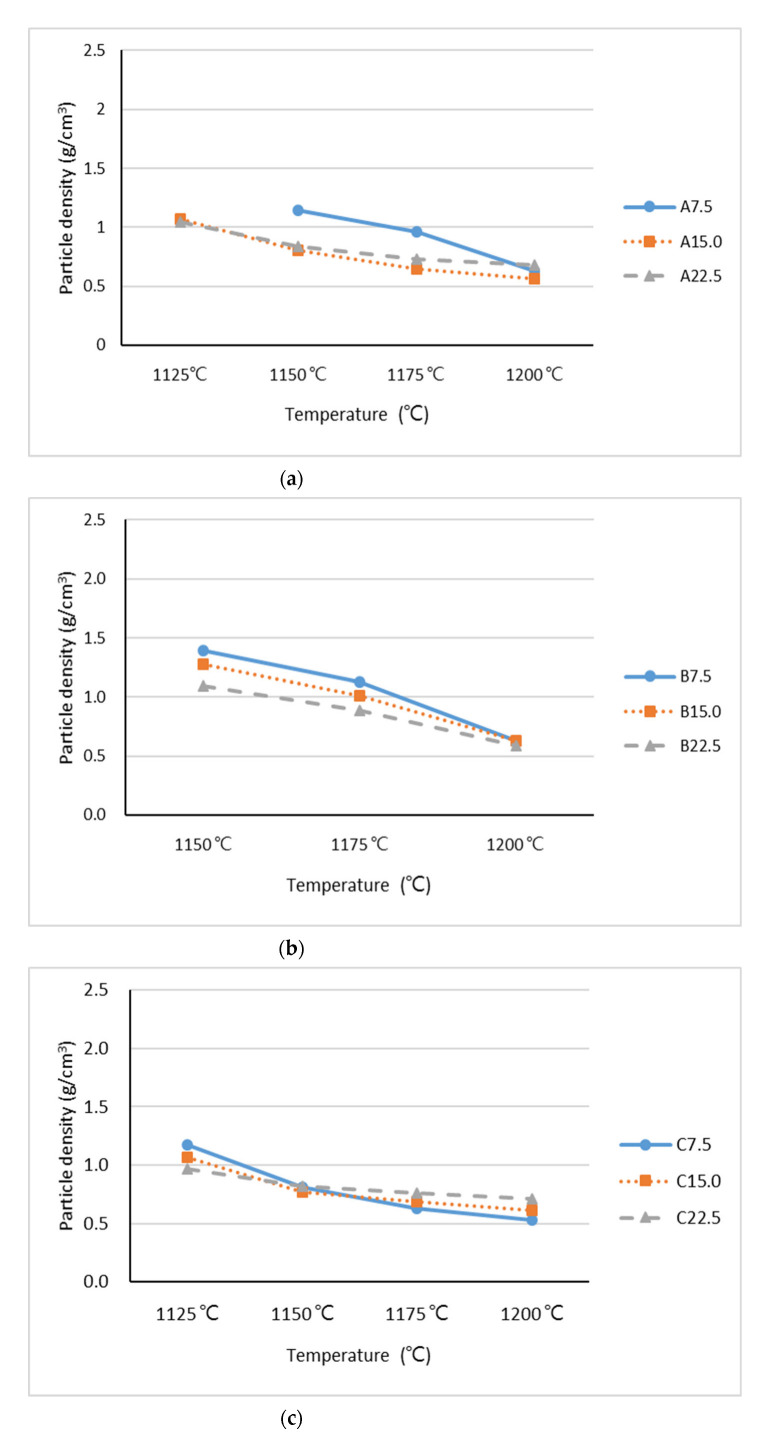
Particle density of lightweight aggregates at Stage I mixed with (**a**) Industrial Sludge A, (**b**) Industrial Sludge B, and (**c**) Industrial Sludge C.

**Figure 4:**
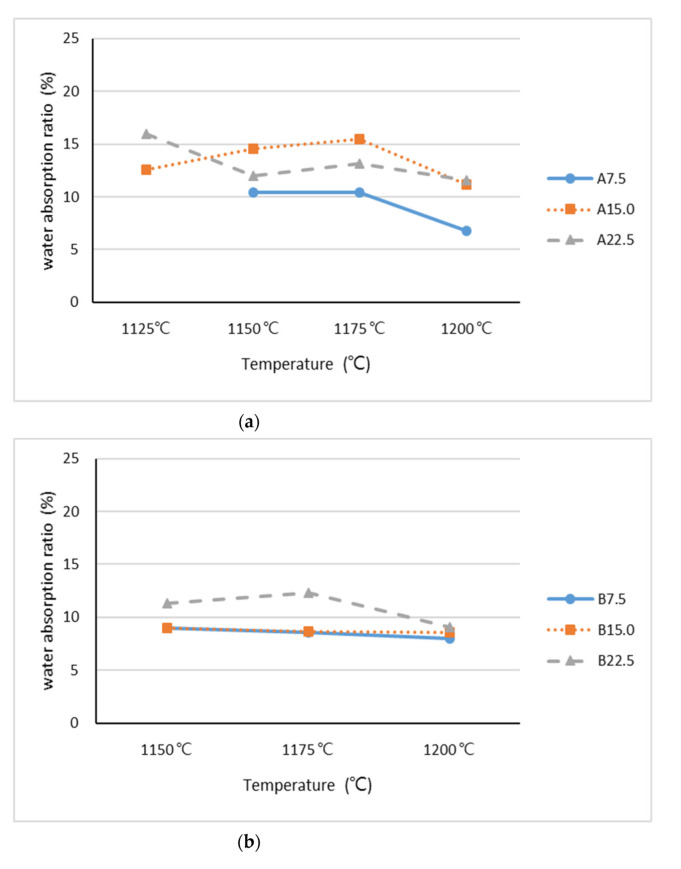

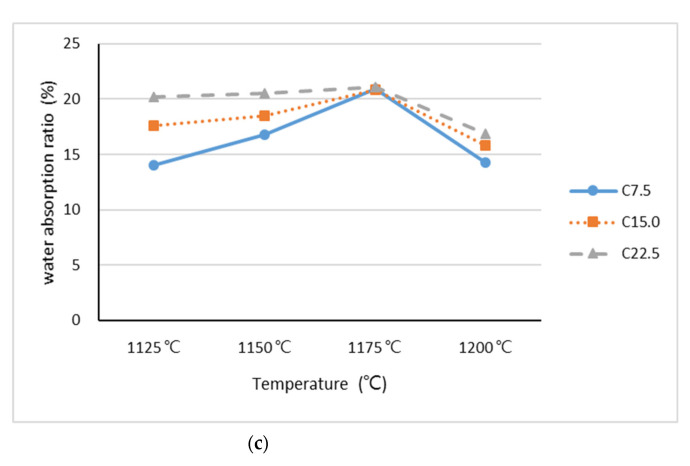
The 24 h water absorption ratios of lightweight aggregates at Stage I mixed with (**a**) Industrial Sludge A, (**b**) Industrial Sludge B, and (**c**) Industrial Sludge C.

According to ACI 213R-14, the properties of lightweight aggregate are significantly affected by shape and surface texture, specific gravity, and water absorption. Hence, we conducted a particle density test, water absorption test, and observation of the shape and surface texture to evaluate the feasibility of the sintered lightweight aggregate for further applications. The Stage I sintering experiments were performed with a fixed preheating temperature and duration and at various sintering temperatures to determine the optimal sintering temperature for the Stage II experiment. The sintering results are presented in Figure 3 and Figure 4. Figure 3 indicates that the particle densities of the industrial sludge lightweight aggregates were between 0.5 and 1.4 g/cm^3^, and that a higher sintering temperature tended to reduce particle density. Producing a lower particle density at a lower sintering temperature is more beneficial from the perspective of reducing energy consumption. In the present study, the lowest sintering temperature capable of producing a particle density of less than 1.0 g/cm^3^ was selected as the optimal sintering temperature; this optimal sintering temperature was the reference for the Stage II experiment. Figure 3a indicates that in the Industrial Sludge A samples, A22.5 and A15 yielded particle densities of less than 1.0 g/cm^3^ at a sintering temperature of 1150 °C. The sample A7.5 could not be sintered to produce lightweight aggregate at a sintering temperature of 1125 °C, probably because the sintering temperature was insufficient. Moreover, sample A7.5 had to be sintered at 1200 °C to produce a particle density less than 1.0 g/cm^3^. Therefore, the Stage II sintering temperatures were 1200° C for the A7.5 sample and 1150 °C for the A15 and A22.5 samples. Figure 3b indicates that the Stage II sintering temperatures were 1200 °C for the samples B7.5 and B15 and 1175 °C for sample B22.5. Figure 3c indicates that the Stage II sintering temperature for the samples C7.5, C15, and C22.5 was set as 1150 °C.

The results pertaining to the 24 h water absorption ratio of the lightweight aggregates in the Stage I experiments are presented in Figure 4. The ranges of water absorption ratios were 6.8%~16% and 8%~12.3% for the lightweight aggregates containing Industrial Sludges A and B, respectively. The water absorption ratios were between 11% and 21.1% among the lightweight aggregates containing Industrial Sludge C. The water absorption ratios of lightweight aggregates that are commonly used in concrete should be less than 20%. Only the lightweight aggregates containing Industrial Sludge C exhibited slightly higher ratios of water absorption, but these were still within the acceptable range.

### 3.4. Results of Stage II Sintering of Industrial Sludge Lightweight Aggregates

**Figure 5:**
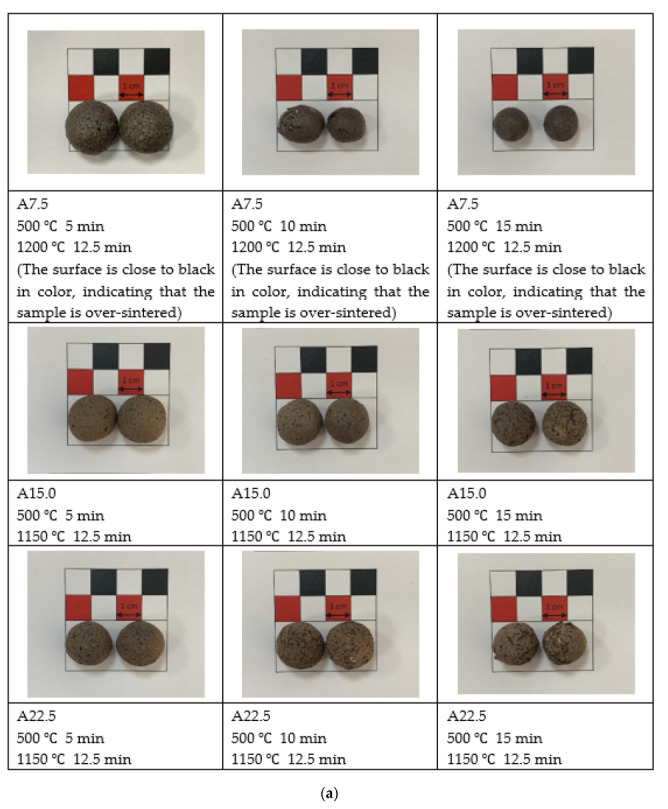

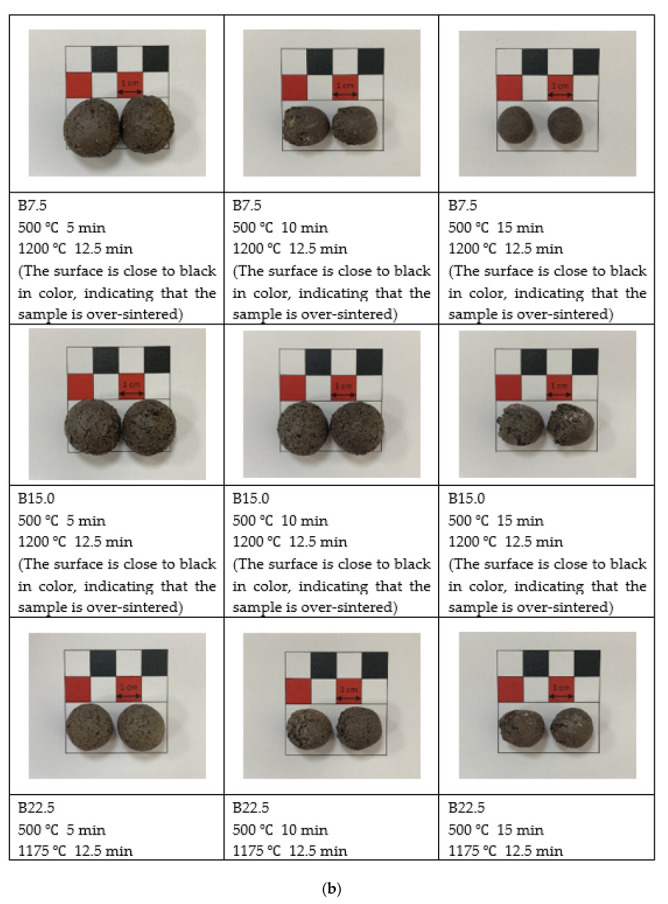

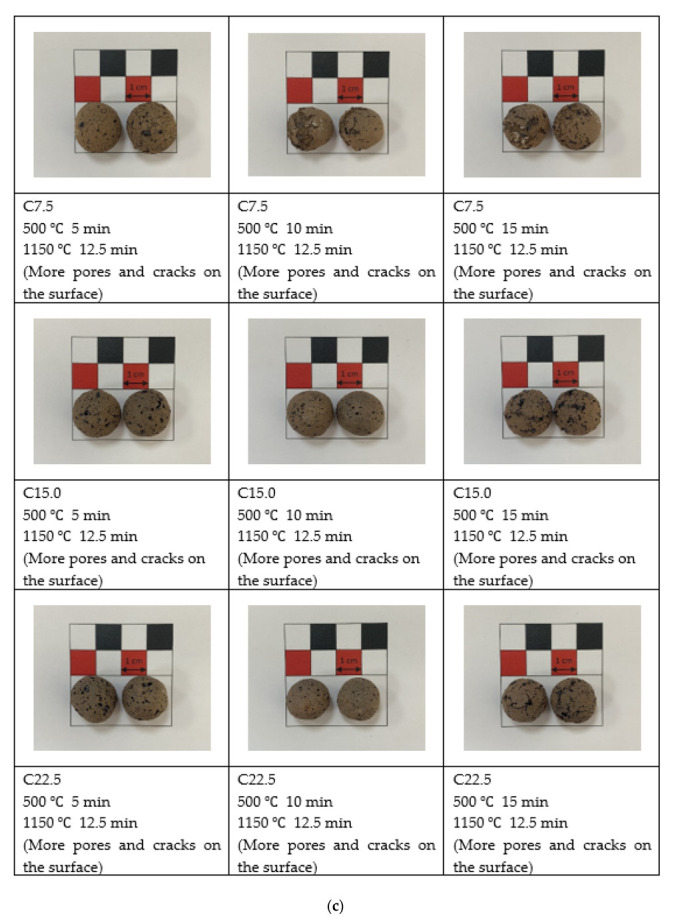
Stage II sintering of lightweight aggregates for (**a**) Industrial Sludge A, (**b**) Industrial Sludge B, and (**c**) Industrial Sludge C.

The conditions of the Stage II sintered experiments are presented in Table 1. The appearances of the sintered samples are depicted in Figure 5. The figures reveal that the aggregates sintered at sintering temperatures of 1150 and 1175 °C were brown in color, whereas those sintered at 1200 °C were closer to black in color. This phenomenon reveals that the lightweight aggregate sintered at 1200 °C was over-sintered. Each set of lightweight aggregates exhibited pores and even cracks on the surfaces. This could be due to the addition of industrial sludge, which has a loss on ignition of between 50% and 70% (Table 2). A sintering temperature of 1150 °C or higher leads to the formation of pores on the surface of the aggregates because of this loss on ignition.

**Figure 6:**
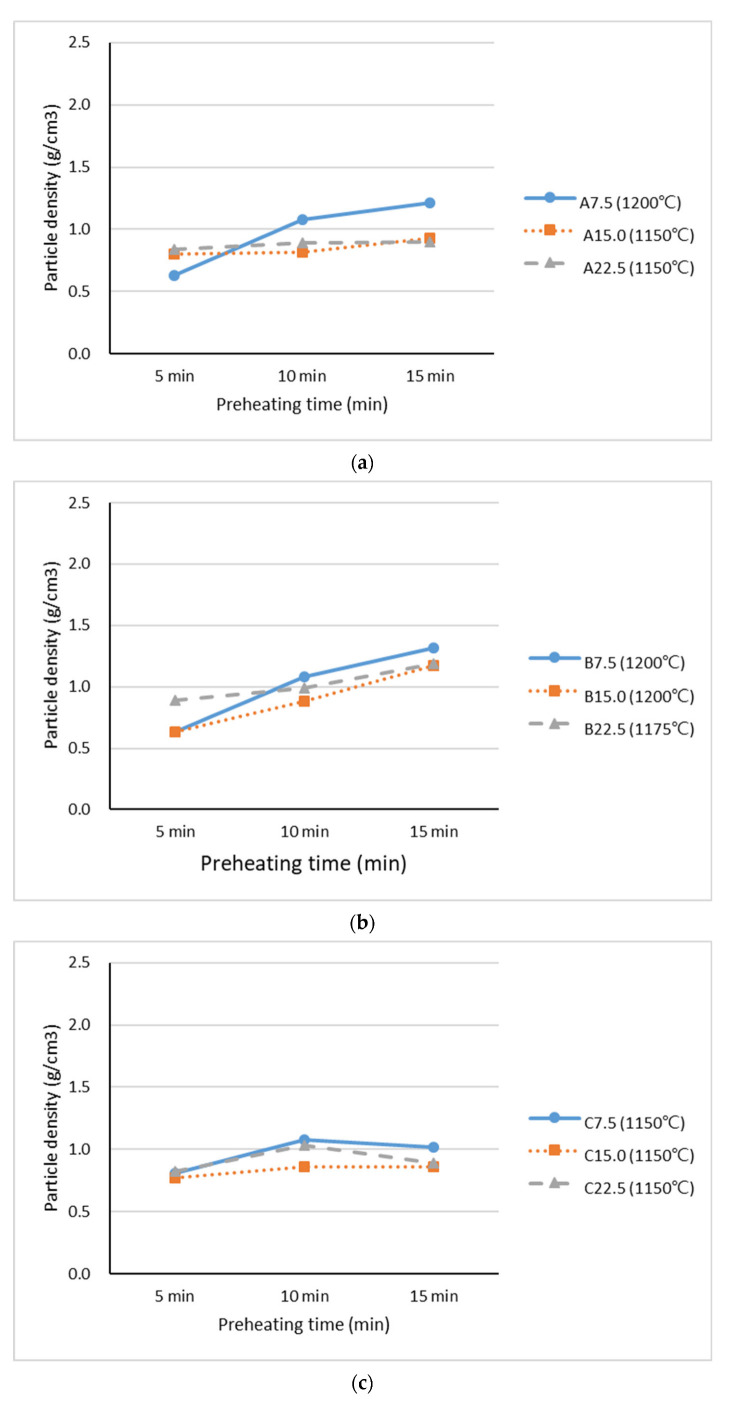
Particle density of lightweight aggregates at Stage II mixed with (**a**) Industrial Sludge A, (**b**) Industrial Sludge B, and (**c**) Industrial Sludge C.

Figure 6 presents the particle density of the lightweight aggregates that contained industrial sludge. Figure 6a,b reveal that the particle densities of Samples A and B increased when the preheating time was increased. The particle density increased from 0.5–0.8 g/cm^3^ to 0.9–1.4 g/cm^3^ as the preheating time increased from 5 to 15 min. A longer preheating time provided more opportunities for the loss of organic matter to occur on ignition. Gases started to escape during the preheating stage when the aggregates were melting, and the aggregates could not produce sufficient gas for an expansion during the sintering stage. Consequently, the aggregates could not expand during the sintering stage, and the particle density increased as the preheating time increased. Industrial Sludge C had an exceedingly high loss on ignition (more than 70%), which could result in a considerable loss within a short preheating period (5 min). As such, increasing the preheating period did not have a significant effect on the particle density, as shown in Figure 6c. The particle density was between 0.8 g/cm^3^ and 0.9 g/cm^3^. According to ACI 213R-14, the particle density of coarse lightweight aggregates corrected to the dry condition is approximately 1/3 to 2/3 that of normal-weight aggregates. Furthermore, in accordance with the specifications of ASTM C330/C330M and C331/C331M, the bulk density of lightweight aggregates for structural concrete should be less than 1120 kg/m^3^ (particle density should be less than 1.80g/cm^3^). The results indicated that the particle densities of the industrial sludge lightweight aggregate were between 0.5 and 1.4, which can meet the requirements of the specifications.

**Figure 7:**
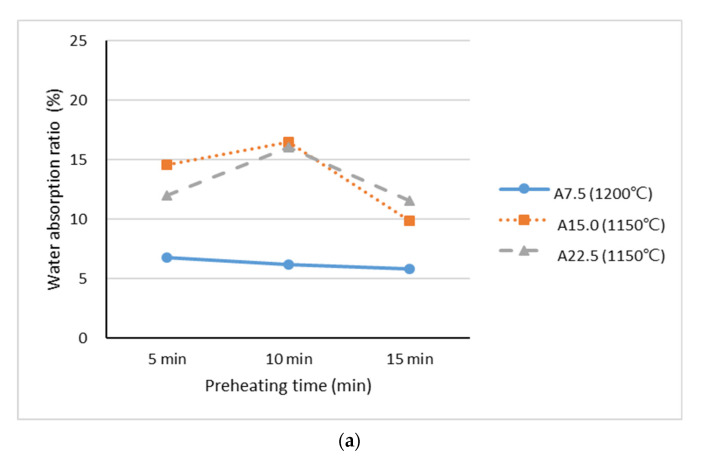

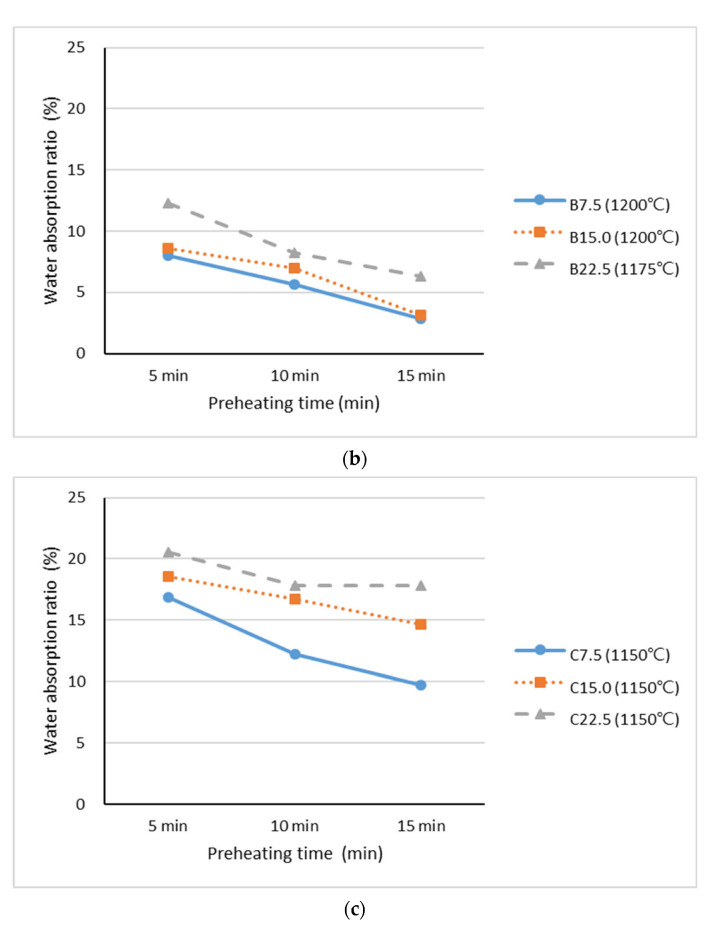
Water absorption ratios of lightweight aggregates at Stage II mixed with (**a**) Industrial Sludge A, (**b**) Industrial Sludge B, and (**c**) Industrial Sludge C.

Lightweight aggregate requires prewetting to maintain as uniform a moisture content as possible before other ingredients are added to the concrete. The proportioned volume of the concrete is then maintained, and slump loss during transport is minimized or eliminated. According to the recommendation of ASTM C94/C94M, the water absorption ratio of lightweight aggregate should preferably be less than 20%. Figure 7 presents the water absorption ratios of the lightweight aggregates made with industrial sludge. The data show that, as the preheating time increased to 15 min, the water absorption of Samples A and B dropped to 3–11%, while the water absorption of Sample C was reduced to 10–18%. The results indicate that increasing the preheating period led to a decrease in the water absorption ratio, which was an inverse trend relative to that for particle density; that is, aggregates with a lower particle density had a higher water absorption ratio. The aggregates sintered at 1200 °C (A7.5, B7.5, and B15) were considered to be of better quality because the water absorption ratios were lower than 10%; this was mainly because the higher sintering temperature resulted in the lightweight aggregates developing finer surface textures and fewer open pores, as shown in Figure 5. The lightweight aggregates containing Industrial Sludge C had higher water absorption ratios of between 10% and 21%, possibly because of the lower sintering temperature (1150 °C) and higher loss on ignition (70%) of Industrial Sludge C; the loss of matter during the sintering process tended to produce open pores on the surfaces of the aggregate, thereby increasing the water absorption ratio. However, according to the standard that the water absorption ratio of lightweight aggregate suitable for use in concrete should less than 20%, except for sample C22.5, which exhibited a water absorption ratio of 21% after 5 min of preheating, the samples met the aforementioned standard defining the usability of lightweight aggregates. According to recommendations, the lightweight aggregates used in general engineering should have a particle density of between 0.5 and 1.8 g/cm^3^ and a water absorption ratio of 20% or less. On the basis of the sintered results in the present study, the addition of WTS from Taiwan containing industrial sludge from Northern, Central, and Southern Taiwan as a secondary ingredient at 7.5%, 15.0%, and 22.5% enabled us to successfully sinter lightweight aggregates for engineering purposes.

**Figure 8:**
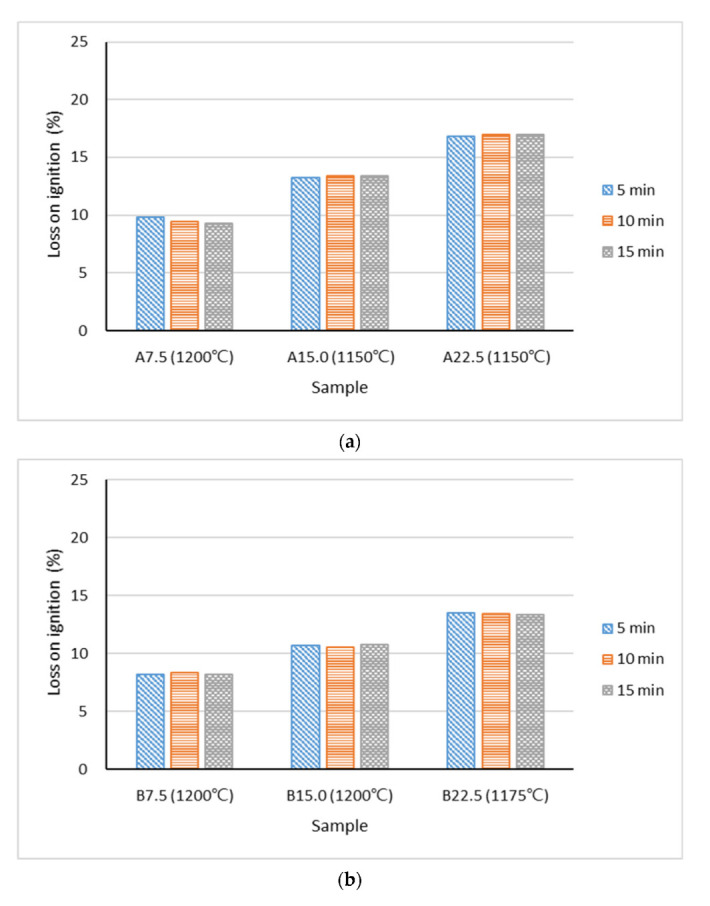

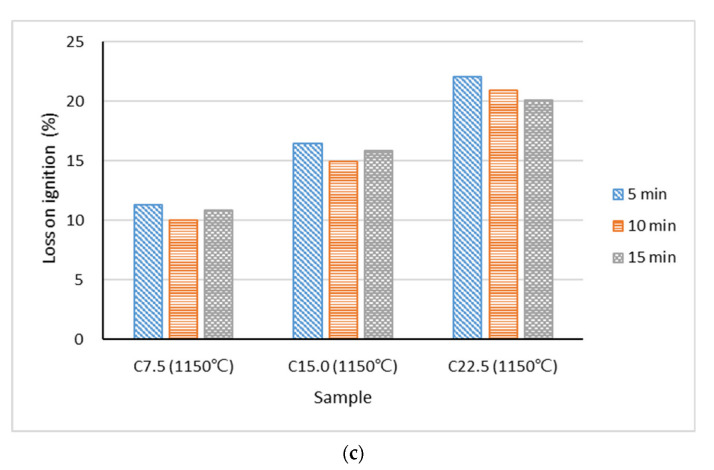
Loss on ignition of lightweight aggregates at Stage II mixed with (**a**) Industrial Sludge A, (**b**) Industrial Sludge B, and (**c**) Industrial Sludge C.

Figure 8 presents the loss on ignition of the lightweight aggregates with various types of industrial sludge. The results indicate that across all sets, the samples with higher proportions of industrial sludge demonstrated greater loss on ignition. The average losses on ignition were 17%, 14%, and 21% for the A22.5, B22.5, and C22.5 samples, respectively. This was mainly because the industrial sludge had a loss on ignition of between 50% and 70% (Table 2), indicating that a large portion of the industrial sludge that was added was burned off. Therefore, loss on ignition among the lightweight aggregates was affected more by the amount of industrial sludge in the mixture and less by the duration of preheating. A greater loss on ignition (10–22%) was observed for all lightweight aggregates containing Industrial Sludge C; this could be explained by the high loss on ignition of Industrial Sludge C (more than 70%). This massive loss on ignition tended to produce open pores on the surfaces of the aggregates (Figure 5c), which increased the water absorption ratio (Figure 7c).

On the basis of the energy required to sinter lightweight aggregates, a greater loss on ignition indicates that a greater amount of raw material was converted into heat energy, which may reduce the energy requirement for sintering lightweight aggregates. Therefore, the use of industrial sludge as a raw material for producing lightweight aggregates and the development of a method for reusing industrial sewage waste may reduce the consumption requirement for sintering lightweight aggregates by leveraging the heat energy released by industrial sludge on ignition, thereby achieving energy-saving and carbon-reduction goals.

## 4. Conclusions

The present study investigated the feasibility of sintered lightweight aggregates using WTS mixed with industrial sludge. The sintering experiment was divided into two stages, and the results are as follows.

1. According to the chemical composition analysis of the industrial sludge, the total combined contents of the chemical components SiO_2_, Al_2_O_3_, and Fe_2_O_3_ were 17.73% in Industrial Sludge A, 15.57% in Industrial Sludge B, and 11.59% in Industrial Sludge C. These amounts are considerably less than the 75% that is recommended by the literature; hence, the industrial sludge could not produce a glassy melt and be independently sintered into lightweight aggregates. Consequently, the industrial sludge was only used as an additional ingredient in the present study.
2. The chemical composition analysis of the WTS revealed that the WTS mixed with industrial sludge at 7.5%, 15.0%, and 22.5% as an additional ingredient had (SiO_2_ + Al_2_O_3_)/fluxing mass ratios that were between 3.5 and 10, which is consistent with the range recommended in the literature for sintering lightweight aggregates. The ternary phase diagrams of the chemical components were also consistent with the recommendations by C.M. Riley, indicating that the ingredients could expand.
3. The sintering results of the light aggregates showed that the particle density of the industrial sludge light aggregates was between 0.5 and 1.4 g/cm^3^, meeting the specification requirements. The water absorption rate was below 21%, which is roughly in line with the recommendations of the specification. When the amount of industrial sludge added was less than 22.5%, the lightweight aggregate could be sintered successfully and was suitable for engineering applications.
4. Every lightweight aggregate exhibited pores and even cracks on the surfaces. This is probably due to the addition of industrial sludge, which has a loss on ignition of between 50% and 70%. A sintering temperature of 1150 °C or higher may lead to the formation of pores on the surface of the aggregates through loss on ignition. Industrial Sludge C had a 71.61% loss on ignition, and this loss on ignition may produce numerous open pores on the surface of the aggregates. In accordance with the observation of the lightweight aggregates’ surface color, sintering at 1200 °C may have been too high, resulting in over-sintering.
5. The particle density of the lightweight aggregates made with industrial sludge increased as the preheating time was increased. The particle density increased from 0.5–0.8 g/cm^3^ to 0.9–1.4 g/cm^3^ as the preheating time increased from 5 to 15 min. Increasing the preheating time probably provided more opportunities for loss of organic matter to occur on ignition. Gases began to escape during the preheating stage as the aggregates were melting, and the aggregates may not have produced sufficient gas for expansion during the sintering stage. The aggregates could not expand during the sintering stage, and the particle density increased as the preheating time was increased. For the samples containing Industrial Sludge C, the preheating time did not appear to have a significant effect on the particle density, probably due to the very high loss on ignition during the shorter preheating time.
6. The results of water absorption tests for the lightweight aggregates revealed that the water absorption tended to decrease as the preheating time was increased. When the preheating time increased to 15 min, the water absorption of Samples A and B dropped to 3–11%, while the water absorption of Sample C was reduced to 10–18%. This trend is the inverse of that for particle density; that is, lightweight aggregates with a lower particle density had higher water absorption.
7. The loss on ignition results revealed that the lightweight aggregates with greater amounts of industrial sludge experienced higher rates of loss on ignition. This was mainly because the loss on ignition of the industrial sludge was between 50% and 70%, indicating that most of the added industrial sludge was burned off.
8. On the basis of the energy required to sinter lightweight aggregates, a greater loss on ignition indicates that a greater amount of raw material was converted into heat energy. Therefore, the use of industrial sludge as a raw material for creating lightweight aggregates and the development of methods for reusing industrial sewage waste may reduce the consumption requirements for sintering lightweight aggregates by leveraging the heat energy released by industrial sludge on ignition, thereby achieving energy-saving and carbon-reduction goals.

## Figures and Tables

**Table 1:** Sintering conditions for Stages I and II.

Stage	Preheating		Sintering	
	Temperature (°C)	Duration (min)	Temperature (°C)	Duration (min)
Stage I	500	5	1125, 1150, 1175, 1200	12.5
Stage II	500	5, 10, 15	Optimal sintering temperature	12.5

**Table 2:** Chemical composition of sludge samples (% by weight).

Chemical Composition	WTS	A	B	C
SiO_2_	58.84	3.57	5.12	2.85
Al_2_O_3_	19.30	11.9	5.73	4.42
Fe_2_O_3_	6.63	2.26	4.72	4.29
Na_2_O	1.15	0.51	1.24	0.42
CaO	1.75	8.85	3.40	3.86
K_2_O	3.55	0.30	0.21	0.34
P_2_O_5_	0.13	8.51	4.34	5.67
Cl	ND	0.26	0.09	0.36
SO_3_	0.37	5.31	13.06	3.74
CuO	0.01	2.31	0.47	0.24
NiO	0.01	0.15	0.15	0.15
MnO	0.09	0.18	-	0.58
MgO	2.24	0.45	1.38	0.07
ZnO	0.02	0.40	7.73	0.30
SrO	0.02	0.13	0.04	0.10
TiO_2_	0.87	0.11	0.58	0.34
ZrO_2_	0.04	0.03	0.04	0.03
Cr_2_O_3_	0.02	0.05	1.23	0.44
Ba	-	-	0.30	-
Br	-	0.01	0.01	0.01
SnO_2_	-	0.28	-	-
Rb_2_O	0.02	-	-	-
F	-	-	-	0.13
Sn	-	-	0.14	0.04
Co_2_O_3_	-	-	0.04	-
PbO	-	0.08	0.04	-
Mo	-	-	-	0.01
Loss on ignition	4.94	54.53	49.94	71.61
Total	100	100	100	100

Notes: Results were determined through X-ray fluorescence analysis. Loss on ignition was calculated on the basis of a temperature of 1000 °C sustained for 1 h. ND indicates that an element point component was less than 0.01% or was 0%.

**Table 3:** Chemical composition of sludge samples (% by weight).

Chemical Composition	WTS	A	B	C
SiO_2_	58.84	3.57	5.12	2.85
Al_2_O_3_	19.30	11.9	5.73	4.42
Fe_2_O_3_	6.63	2.26	4.72	4.29
SUM	84.77	17.73	15.57	11.59

**Table 4:** The chemical component analysis of fluxing agents (% by weight).

Chemical Component	WTS	A	B	C
Fe_2_O_3_	6.63	2.26	4.72	4.29
K_2_O, Na_2_O	4.70	0.81	1.45	0.76
CaO, MgO, FeO	3.99	9.30	4.78	3.93
SUM	15.32	12.37	10.95	8.98

**Table 5:** The chemical component analysis of the (SiO_2_ + Al_2_O_3_)/fluxing ratio (% by weight).

Chemical Component	WTS	Industrial Sludge A			Industrial Sludge B			Industrial Sludge C		
		A7.5	A15.0	A22.5	B7.5	B15.0	B22.5	C7.5	C15.0	C22.5
SiO_2_	63.0	59.2	55.4	51.7	59.6	56.1	52.7	60.0	57.0	54.1
Al_2_O_3_	20.7	22.3	24.0	25.6	21.1	21.6	22.1	21.1	21.5	21.9
Fluxing	16.4	18.5	20.6	22.7	19.3	22.2	25.1	18.9	21.5	24.0.
(SiO_2_ + Al_2_O_3_)/Fluxing	5.1	4.4	3.9	3.4	4.2	3.5	3.0	4.3	3.7	3.2

## Data Availability

Not applicable.
